# Feather mites (Acari, Astigmata) from Azorean passerines (Aves, Passeriformes): lower species richness compared to European mainland

**DOI:** 10.1051/parasite/2015009

**Published:** 2015-02-11

**Authors:** Pedro Rodrigues, Sergey Mironov, Oldrich Sychra, Roberto Resendes, Ivan Literak

**Affiliations:** 1 CIBIO, Centro de Investigação em Biodiversidade e Recursos Genéticos, InBIO Laboratório Associado, Pólo dos Açores, Universidade dos Açores 9501-801 Ponta Delgada Portugal; 2 Zoological Institute, Russian Academy of Sciences 199034 Saint Petersburg Russia; 3 Faculty of Veterinary Hygiene and Ecology, Department of Biology and Wildlife Diseases, University of Veterinary and Pharmaceutical Sciences Brno Palackeho 1-3 61242 Brno Czech Republic

**Keywords:** Passeriformes, Feather mites, Host-parasite associations, Biodiversity, Prevalence, Azores

## Abstract

Ten passerine species were examined on three islands of the Azores (North Atlantic) during 2013 and 2014 in order to identify their feather mite assemblages. We recorded 19 feather mite species belonging to four families of the superfamily Analgoidea (Analgidae, Proctophyllodidae, Psoroptoididae and Trouessartiidae). A high prevalence of feather mite species was recorded on the majority of the examined host species. Only three passerine species (*Sylvia atricapilla, Regulus regulus* and *Serinus canaria*) presented the same full complex of mite species as commonly occurs in the plumage of their closest relatives in continental Europe. *Passer domesticus* presented the same limited fauna of feather mites living in the plumage as do its co-specifics in continental Europe. *Carduelis carduelis* bears the same feather mite species as do most of its continental populations in Europe, but it lacks one mite species occurring on this host in Egypt. *Turdus merula, Pyrrhula murina* and *Fringilla coelebs* are missing several mite species common to their continental relatives. This diminution could be explained by the founder effect, whereby a limited number of colonizing individuals did not transport the full set of feather mite species, or by the extinction of some mite species after initially having reached the Azores. The only individual of *Motacilla cinerea* sampled in this study presented a new host record for the mite species *Trouessartia jedliczkai*.

## Abstract (fr)

Dix espèces de passereaux ont été examinées sur trois îles des Açores (Atlantique Nord) en 2013 et 2014 afin d’identifier leurs assemblages d’acariens plumicoles. Nous avons trouvé 19 espèces d’acariens plumicoles appartenant à quatre familles de la superfamille Analgoidea (Analgidae, Proctophyllodidae, Psoroptoididae et Trouessartiidae). Une prévalence élevée d’espèces d’acariens plumicoles a été trouvée sur la majorité des espèces hôtes examinés. Seules trois espèces de passereaux (*Sylvia atricapilla*, *Regulus regulus* et *Serinus canaria*) présentaient le même complexe complet d’espèces d’acariens qui existe généralement en Europe continentale dans le plumage de leurs parents les plus proches. *Passer domesticus* présentait la même faune limitée d’acariens plumicoles vivant dans le plumage que ses conspécifiques en Europe continentale. *Carduelis carduelis* hébergeait les mêmes espèces d’acariens plumicoles que la plupart de ses populations continentales en Europe, mais n’avait pas une espèce d’acarien qui vit sur cet hôte en Égypte. *Turdus merula*, *Pyrrhula murina* et *Fringilla coelebs* n’hébergeaient pas plusieurs espèces d’acariens communes à leurs parents continentaux. Cette diminution pourrait s’expliquer par l’effet fondateur, dans lequel un nombre restreint d’individus colonisateurs ne transportent pas l’ensemble des espèces de leurs acariens plumicoles, ou par l’extinction de certaines espèces d’acariens après avoir atteint les Açores. Le seul individu de *Motacilla cinerea* échantillonné dans cette étude a présenté une nouvelle mention d’hôte pour l’espèce d’acarien *Trouessartia jedliczkai*.

## Introduction

Feather mites comprise a group of psoroptidian mites (Acariformes: Astigmata) with roughly 2600 currently accepted species arranged in 36–38 families and two superfamilies, Analgoidea and Pterolichoidea [16, 28, 30, 34, 36, 45]. These mites are parasites or commensals permanently living on birds, never leaving their hosts, and highly specialized to numerous and quite different microhabitats on the bird body [7, 16]. Not all mites attributed to these superfamilies and named feather mites are true inhabitants of the plumage. While the majority of species indeed inhabit different types of plumage (flight feathers, body and down feathers), representatives of some families live in the internal cavities of the feather quill, on the surface of skin, in subcutaneous layers of the skin, and in the nasal cavities [7]. Feather mites are known from all extant orders of birds, including penguins (as recently determined), and species bear a complex of specific feather mite species living in different microhabitats on the host body [16, 29, 37].

Studies focusing on feather mite diversity have been conducted in most mainland countries of the European continent (see Mironov [26, 27] for major references). Such studies are lacking for the Atlantic islands and in particular for the archipelago of the Azores. This archipelago is situated towards the middle of the Atlantic Ocean. Its avifauna encompasses 40 breeding species, 14 of which are passerines and include the endemic *Pyrrhula murina* (Godman, 1866) and 7 other endemic subspecies [39]. To date, only two feather mite species have been reported from two procellariiform hosts from the Azores [48, 49]. In addition, the first study focusing upon arthropod ectoparasites associated with passerine birds on this archipelago was carried out only recently [21]. The study revealed that the assemblage on *Sylvia atricapilla gularis* (Alexander, 1898) is composed of common chewing louse species to this passerine in Europe and the prevalence of the parasites was much higher in the Azorean host population than in the mainland populations.

In the present paper, we report for the first time the presence of 19 feather mite species belonging to four families found on 10 different passerine hosts from three islands of the Azores. We also compare the feather mite complexes found on Azorean passerines with those known on these avian species or their closest relatives in continental Europe based on reference data, since host species that colonize new regions, such as isolated islands, often lose parasite species [11, 22, 52], but this assumption has never been tested on feather mites before.

## Materials and methods

Our study was focused on three islands of the Azores archipelago, situated in the Atlantic Ocean between the latitudes 36°55′ and 39°43′ North and the longitudes 24°46′ and 31°16′ West: São Miguel (37°48′35ʺ N 25°12′51ʺ W), Santa Maria (36°58′58ʺ N 25°05′27ʺ W) and Graciosa (39°03′05ʺ N 28°00′51ʺ W). It is roughly 1500 km from continental Europe and 1900 km from continental North America. The Azores encompass nine islands of recent volcanic origin (between 0.25 and 8 My old) that are spread along a northwest-southeast line over more than 600 km [13].

A total of 253 passerines belonging to 10 of the 14 Passeriforme breeding species on the Azores (Table 1) were captured using mist nets within different habitats on the three islands of this study. Passerines were sampled at various locations on São Miguel during April, June and July 2013 and June 2014, and on Santa Maria and Graciosa during September 2013. Bird individuals were identified, ringed and immediately released into the wild after removing one primary remex and/or one rectrix feather sample containing feather mites as detected by the naked eye. The feathers removed were preserved in 96% ethanol for future processing. Under laboratory conditions, mite specimens were then mounted on microslides in Faure’s medium according to a standard technique for small acariform mites [12]. Mites were identified by one of the authors (SM) using a Leica DM 5000B light microscope with differential interference contrast illumination. Specimens mounted on slides are deposited at the Zoological Institute of the Russian Academy of Sciences (Saint Petersburg, Russia).

**Table 1.** Passerine species examined per island.

Bird species	Family	Number of examined birds			Total
		São Miguel	Santa Maria	Graciosa	
*Carduelis carduelis parva* (Tschusi, 1901)	Fringillidae	2	0	0	2
*Erithacus rubecula* (Linnaeus, 1758)	Muscicapidae	9	6	3	18
*Fringilla coelebs moreletti* (Pucheran, 1859)	Fringillidae	26	14	62	102
*Motacilla cinerea patriciae* (Vaurie, 1957)	Motacillidae	1	0	0	1
*Passer domesticus* (Linnaeus, 1758)	Passeridae	2	0	1	3
*Pyrrhula murina* (Godman, 1866)	Fringillidae	18	*	*	18
*Regulus regulus azoricus* (Seebohm, 1883)	Regulidae	6	*	*	6
*Regulus regulus sanctaemariae* (Vaurie, 1954)	Regulidae	*	10	*	10
*Serinus canaria* (Linnaeus, 1758)	Fringillidae	19	10	0	29
*Sylvia atricapilla gularis* (Alexander, 1898)	Sylviidae	11	23	5	39
*Turdus merula azorensis* (Hartert, 1905)	Turdidae	11	12	2	25
Total		105	75	73	253

* Species do not breed on this island.

## Results

From the 253 passerines sampled (Table 1), feather mites were not found on the primary and tail feathers of 14 birds, namely, on one individual of *Fringilla coelebs moreletti* (Pucheran, 1859) from Graciosa, and on two individuals of *Passer domesticus* (Linnaeus, 1758) and 11 *Pyrrhula murina* from São Miguel. The other 239 (94%) individuals sampled hosted one, two or three different feather mite species. In total, 19 feather mite species representing five genera and four families (Analgidae, Psoroptoididae, Proctophyllodidae and Trouessartiidae) were identified (Table 2). The prevalence of recorded feather mite species was high, reaching 100% in some species. With the exception of *Analges passerinus*, which was recorded on three different passerine hosts, all the other determined feather mite species were found on only one host. The recorded feather mite species, their host(s) and their prevalence on each host species are shown in Table 2. All feather mite species found constitute the first such records for the Azores.

**Table 2.** Host species and prevalence of identified avian feather mites.

Bird species	Feather mite species	Family	Number of parasitized birds; prevalence (%)*		
			São Miguel	Santa Maria	Graciosa
*Carduelis carduelis*	*Analges passerinus* (Linnaeus, 1758)	Analgidae	1	–	–
	*Proctophyllodes pinnatus* (Nitzsch, 1818)	Proctophyllodidae	1	–	–
*Erithacus rubecula*	*Trouessartia rubecula* Jablonska, 1968	Trouessartiidae	9; 100%	6; 100%	2
	*Proctophyllodes rubeculinus*	Proctophyllodidae	1; 11.1%	1; 16.7%	2
*Fringilla coelebs*	*Monojoubertia microphylla* Robin, 1877	Proctophyllodidae	25; 96.2%	14; 100%	61; 98.4%
	*Analges passerinus* (Linnaeus, 1758)	Analgidae	1; 3.9%	0; 0%	3; 4.8%
*Motacilla cinera patriciae*	*Trouessartia jedliczkai* (Zimmermann, 1894)	Trouessartiidae	1	–	–
*Passer domesticus*	*Proctophyllodes troncatus* Robin, 1877	Proctophyllodidae	0	–	1
*Pyrrhula murina*	*Mesalgoides pyrrhulinus* Mironov, 1997	Psoroptoididae	6; 33.3%	–	–
	*Analges macropus* Zimmermann, 1894	Analgidae	6; 33.3%	–	–
*Regulus regulus azoricus*	*Trouessartia reguli* Mironov, 1983	Trouessartiidae	6; 100%	–	–
	*Proctophyllodes reguli* Gaud, 1957	Proctophyllodidae	1; 16.7%	–	–
*Regulus regulus sanctaemariae*	*Trouessartia reguli* Mironov, 1983	Trouessartiidae	–	10; 100%	–
	*Proctophyllodes reguli* Gaud, 1957	Proctophyllodidae	–	9; 90%	–
*Serinus canaria*	*Proctophyllodes serini* Atyeo & Braasch, 1966	Proctophyllodidae	17; 89.5%	10; 100%	–
	*Analges passerinus* (Linnaeus, 1758)	Analgidae	12; 63.2%	1; 10%	–
	*Mesalgoides* sp.	Psoroptoididae	1; 5.3%	3; 30%	–
*Sylvia atricapilla gularis*	*Trouessartia bifurcata* (Trouessat, 1884)	Trouessartiidae	10; 90.9%	19; 82.6%	3; 60%
	*Analges spiniger* Giebel, 1841	Analgidae	3; 27.3%	0; 0%	0; 0%
	*Proctophyllodes sylviae* Gaud, 1957	Proctophyllodidae	2; 18.2%	12; 52.2%	3; 60%
*Turdus merula azorensis*	*Analges turdinus* Mironov, 1985	Analgidae	1; 9.1%	0; 0%	0
	*Proctophyllodes weigoldi* Vitzthum, 1922	Proctophyllodidae	8; 72.7%	4; 33.3%	1
	*Trouessartia incisa* Gaud, 1957	Trouessartiidae	9; 81.8%	9; 75%	2

* Calculated only when at least five birds of a given species were examined on the given island.

## Discussion

The present study of feather mites on passerines of the Azores is the first step towards identifying the feather mite communities on the Azores avifauna and understanding their origin, phylogeographic distribution and host-parasite relationships with avian hosts.

All feather mite species recorded in the course of this study belong to the four most abundant families of these mites occurring on passerines. Among them, mites of the families Proctophyllodidae and Trouessartiidae are predominantly associated with passerines, while representatives of the families Analgidae and Psoroptoididae are associated with a wide spectrum of bird orders and only some of their genera are restricted to passerines [16, 36]. Mites of the family Proctophyllodidae are the most typical inhabitants of the ventral side of wing and tail feathers; the Trouessartiidae are usually located on the dorsal side of wing and tail feathers; the Analgidae are most typically inhabitants of the downy feathers of the body; and the Psoroptoididae can inhabit the downy feathers, the greater coverts of the wings and even basal areas of primaries and tail feathers [7, 25].

Regarding the content of parasite assemblages of Azorean passerine hosts, only three species examined in this study – *Sylvia atricapilla, Regulus regulus* (Linnaeus, 1758) and *Serinus canaria* (Linnaeus, 1758) – presented the same and apparently full complex of mite species commonly occurring in the plumage of these hosts or their closest relatives on mainland Europe [1, 4, 19, 24, 26, 27, 43]. These feather mite species complexes had been expected to occur inasmuch as these hosts colonized the Azores quite recently, i.e. within the last 0.7 million years [8, 9, 41]. It is also possible to surmise that the colonizing populations of these birds had borne the full set of feather mites and during the time since colonization of the Azores none of the mite species has become extinct.

*Sylvia atricapilla* commonly bears three species. Among these, *Proctophyllodes sylviae* is a monoxenous inhabitant of this host, *Analges spiniger* also occurs on other species of the genus *Sylvia* Scopoli, 1769 and *Trouessartia bifurcata* is known on *Sylvia* and *Acrocephalus* Naumann and Naumann, 1811 [1, 24, 43].

Each of the two subspecies of *Regulus regulus* (from São Miguel and Santa Maria) sampled during this study bore two species of feather mites that are specific to goldcrests of the genus *Regulus* Cuvier, 1800 [1, 26, 27].

The wild form of *Serinus canaria* is endemic to Macaronesia (Azores, Madeira and Canary Islands). Three mite species recorded on this host are the same as those known from the closely related species *Serinus serinus* (Linnaeus, 1766), which is distributed in continental Europe [26]. Among these species, *Proctophyllodes serini* is specific to the genus *Serinus* Koch, 1816, while *Analges passerinus* is widely distributed on European species of the family Fringillidae [19, 24, 26]. A *Mesalgoides* species found on *S. canaria* and on *S. serinus* in the mainland of Europe (Mironov, unpublished data) is supposed to be a species new to science.

It should be mentioned that only exceptionally domestic canaries (*S. canaria domestica*) have been examined for the presence of feather mites [10, 46]. Schmäschke et al. [46] examined seven captive domestic canaries and found *Proctophyllodes serinii, Analges passerinus* and *Strelkoviacarus* sp. (Analgidae) on four, one and one birds, respectively. The finding of *Strelkoviacarus* sp. on domesticated canaries may be the result of transfer of this mite from wild passerines, because mites of the genus *Strelkoviacarus* (and likely many feather mite species of the family Epidermoptidae) are not typical feather mite species, in that they live on the skin and are able to disperse between different bird species by means of phoresy on hippoboscid flies of the genera *Ornithomyia* Latreille, 1802, *Lynchia* Williston, 1890 and many others specialized to birds (Diptera: Hippoboscidae) [18]; additionally, this mite was never found on wild canaries.

*Carduelis carduelis parva* (Tschusi, 1901) was introduced to the Azores during the 18th century [39]. The goldfinch was represented in our samples by only a single individual and displayed the same complex of species occurring on this host on the European mainland [19, 24, 26, 27]. *Proctophyllodes pinnatus* also occurs on some nearest species of the genus *Carduelis*, while *A. passerinus* is widely distributed on Eurasian passerines of the family Fringillidae. It should be noted, however, that *C. carduelis* has also been reported parasitized by a monoxenous species *Mesalgoides travei* Shereef and Rakha, 1981 in Egypt [47]. Since, despite frequent examination of *C. carduelis* for feather mites, the latter mite species has never been recorded elsewhere than in Egypt, we hypothesize that all European populations of *C. carduelis*, including the Azorean representatives, lack this species.

*Erithacus rubecula* (Linnaeus, 1758), also a recent colonizer of the archipelago [40], bears only two of the three common species that occur on this host in mainland Europe – *P. rubeculinus* and *T. rubecula*; both are specific inhabitants of this passerine [1, 43]. *Analges unidentatus* (Berlese, 1886), the third species known from *E. rubecula*, is distributed on muscicapids of the genera *Muscicapa* Brisson, 1760, *Ficedula* Brisson, 1760, *Phoenicurus* Forster, 1817 and *Erithacus* Cuvier, 1800 and it has been recorded only in north-western Russia [26].

*Turdus merula azorensis* (Hartert, 1905) presented three of the five species known from the plumage of this species on continental Europe. *P. weigoldi* and *A. turdinus* are common species occurring on several other *Turdus* species in the Old World [1, 26], while *Trouessartia incisa* is known only from *T. merula* in Europe and Morocco [14, 43]. The two other species, *P. musicus* Vitzthum, 1922 and *Montesauria merulae* Gaud, 1957, known also to be associated with various species of the genus *Turdus* [1, 14], are probably not present in the population of *T. merula* in the Azores.

*Pyrrhula murina*, endemic from São Miguel Island, displayed two feather mite species, *Mesalgoides pyrrhulinus* and *Analges macropus*, previously known to be specific to *Pyrrhula pyrrhula* [15, 24, 26, 27]. Quite surprising is the absence in this bird of *Proctophyllodes simillimus* Černý, 1971, which is common to the closely related species *P. pyrrhula* [6, 26, 27].

*Fringilla coelebs moreletti* displayed two of the three species occurring on the mainland populations of this host in Europe. *Monojoubertia microphylla* is a monoxenous inhabitant of this host, while *A. passerines* is associated with various Eurasian fringillids of the genera *Fringilla, Carduelis, Acanthis* and *Spinus* [2, 4, 19, 26, 27]. *Pteronyssoides striatus* (Robin, 1877) (Pteronyssidae), a monoxenous inhabitant of the European *F. coelebs* [25, 27], presumably is not present on this host in the Azores.

These three hosts, *Turdus merula*, *Pyrrhula murina* and *Fringilla coelebs*, are the oldest passerine species to have colonized the archipelago, doing so more than 1 million years ago [38, 42, 51]. One possible explanation for the absence of some common feather mites on these passerines in the Azores could be the founder effect inherent to the colonization processes of isolated islands, called “missing the boat” by Macleod et al. [22], in which colonizing hosts may reach the islands carrying only a subset of their respective native feather mites assemblages [20, 32, 44]. On the other hand, it is also possible that the aforementioned mite species simply became extinct just after colonization of the Azores, phenomena called “drowning upon arrival” [22], due to some unfavourable environmental conditions, such as climate factors, which are known to influence the prevalence of feather mites on hosts [23]. As indicated above, such relatively new invaders to the Azores as *Sylvia atricapilla, Regulus regulus* have completely retained their native continental fauna.

The only individual of *Motacilla cinerea patriciae* (Vaurie, 1957) sampled during this work presented only one feather mite species, *Trouessartia jedliczkai*. This is a relatively common species from *M. alba* (Linnaeus, 1758) and *M. flava* (Linnaeus, 1758) [5, 17, 26, 43], but it has never been recorded before on *M. cinerea*. More samples are required in order to elucidate whether *M. cinerea* in the Azores bears feather mite species of the families Proctophyllodidae, Trouessartiidae and Analgidae as *Motacilla* spp. in continental Europe.

*Passer domesticus*, introduced to the Azores in 1960 [39], has a quite limited fauna of feather mites living in its plumage even in Europe, in almost all studies consisting of the single species *Proctophyllodes troncatus* [3, 4, 17, 19, 26, 27]. This species is also common in Europe on other species of the genus *Passer*. Although only a single *P. domesticus* individual occurs in our samples, it is possible to state that its population, at least on Graciosa Island, has retained the original feather mite fauna from the mainland.

Thus, our study has shown that a number of passerine birds which colonized the Azores lack some of the feather mites occurring in the corresponding complexes of their populations or those of closely related species in continental Europe, which could have been caused by incomplete feather mite fauna carried by the individuals that colonized the Azores (i.e. the founder effect), or this could be due to the extinction of some mite species caused by some climatic conditions in this colonized territory.

The high prevalence of feather mite species recorded on the majority of hosts in this study could be due to the fact that all the passerine species sampled are sedentary and do not migrate [39]; which could also be the reason for the presence of lower species richness compared to the European mainland, since it is known that migratory birds are exposed to a wider assortment of mites than are non-migratory birds such as the Azorean species [31, 50]. Another possible explanation for the high prevalences of feather mites on the majority of the hosts could lie in the fact that some of these species regularly form flocks during winter, and it is known that flocking species have higher mite prevalences than do solitary species [35]. Feather mites tend to be transmitted between individuals of the same group (flock) as well as from parents to offspring [33].
